# Hepatic infarction occurred after ^125^I particle stent treatment for hepatocellular carcinoma with portal vein tumor thrombus: A case report

**DOI:** 10.1007/s00432-024-05826-y

**Published:** 2024-06-17

**Authors:** Jiemin Yang, Yang Qin, Zhongyuan Lv, Qingqing Xiao, Ying Miao, Huiping Huang, Bing Wei, Jingsong Mao

**Affiliations:** 1 Department of Vascular Intervention, Affiliated Hospital of Guilin Medical University, Guilin, 541001 Guangxi China; 2 Guilin Medical University, Guilin, 541001 Guangxi China; 3 Department of Graduation, Affiliated Hospital of Guilin Medical University, Guilin, 541001 Guangxi China

**Keywords:** Hepatocellular carcinoma, Portal vein tumor thrombus, Portal vein stent implantation, Stent thrombus, Hepatic infarction

## Abstract

**Background:**

Hepatic infarction is a rare liver condition. The purpose of this study is to report a case of hepatic infarction caused by thrombus formation following portal vein stent implantation in a patient with hepatocellular carcinoma and portal vein tumor thrombus, and to explore the underlying causes.

**Case report:**

The patient in this study was a 52-year-old male admitted with diffuse hepatocellular carcinoma involving the right lobe and portal vein tumor thrombus. After undergoing portal vein stent implantation and ^125^I particle strand implantation treatment, the portal vein was patent, and the pressure decreased. However, multiple instances of hepatic artery chemoembolization combined with targeted immunotherapy resulted in gradual reduction in the diameter of the hepatic artery and affecting hepatic arterial blood flow. Two months post-stent implantation, thrombus formation within the stent was noted, and the patient’s condition did not improve with anticoagulant therapy, as evidenced by follow-up CT scans showing an increase in thrombi. Six months later, the patient suffered from gastrointestinal bleeding and, despite emergency esophagogastric variceal ligation and hemostatic treatment, developed hepatic parenchymal infarction and liver function failure.

**Conclusions:**

We reveal the underlying cause is that (1) thrombus formation within the portal vein stent, leading to portal vein embolism and obstructed blood flow due to exacerbate portal hypertension after various treatments; and (2) the effect of hepatic artery chemoembolization, immunotherapy, and targeted therapy on tumor angiogenesis, causing reduced hepatic artery diameter and impaired arterial blood flow. These factors disrupt the liver’s dual blood supply system, ultimately contributing to hepatic infarction. To our knowledge, this is the first report of hepatic infarction as a complication following portal vein stent implantation for hepatocellular carcinoma with portal vein tumor thrombus, and it holds significant reference value for guiding the treatment of hepatocellular carcinoma with concurrent portal vein tumor thrombus in a clinical setting.

## Introduction

Primary liver cancer is a common digestive system disease that poses a threat to human health. When liver cancer cells invade the portal vein and form tumor thrombus, it can further lead to severe complications such as ascites, gastrointestinal bleeding, and liver function failure. Most patients with hepatocellular carcinoma with portal vein tumor thrombus have lost the opportunity for surgical treatment. The main treatment options for such patients currently include portal vein stent implantation, intra-arterial radiation therapy, laser radiofrequency ablation, and hepatic artery chemoembolization, which are palliative treatments ((Li et al. 2020a; Yu et al. 2017; Chuan-Xing et al. 2011)). Among them, portal vein stent implantation can quickly restore blocked portal vein blood flow, reduce portal hypertension, improve liver function, and reduce the risk of variceal bleeding ((Li et al. 2020a; Yu et al. 2017; Luo et al. 2016)). Continuous irradiation with ^125^I particles can inhibit tumor cell proliferation and induce apoptosis, while low-dose radiation can suppress neointimal hyperplasia after stent implantation, prolong stent patency, and inhibit the development of tumor thrombus (Chuan-Xing et al. 2011; Luo et al. 2016). In particular, the combination of portal vein stent implantation and ^125^I particle strand implantation exposes the portal vein tumor thrombus directly to radiation, thus controlling tumor progression. Therefore, portal vein stent implantation combined with ^125^I particle strand implantation has become a safe and effective treatment for patients with hepatocellular carcinoma and portal vein tumor thrombus (Zhang et al. 2016; Lu et al. 2017).

The occurrence of hepatic infarction as a consequence of portal vein stent surgery is exceedingly rare, and to our knowledge, with no documented case reports to date. This study presents the first report of hepatic infarction following portal vein stent implantation. In this report, the patient underwent portal vein stent implantation combined with ^125^I particle strand implantation, and subsequently received four sessions of hepatic artery chemoembolization (D-TACE), immunotherapy, and targeted therapy for the primary liver tumor. The aforementioned treatment regimen significantly inhibited tumor angiogenesis and reduced the size of the primary lesion. However, two months after the stent implantation procedure, thrombus formation was observed within the portal vein stent, and despite anticoagulant therapy, the treatment outcome was unsatisfactory. Follow-up CT scans revealed an increasing number of thrombi within the stent. Furthermore, six months after the procedure, the patient endured esophageal variceal rupture and bleeding. Despite endoscopic variceal ligation and a two-week course of hemostatic treatment, the patient suddenly developed severe upper abdominal pain accompanied by refractory hypotension and liver function failure. Through analyzing the patient’s clinical manifestations, imaging examinations, diagnosis, and subsequent consequences, we indicate that hepatic infarction in this case is primarily caused by two factors: (1) thrombus formation within the portal vein stent, leading to portal vein embolism and obstructed blood flow due to exacerbated portal hypertension after various treatments; and (2) the effect of hepatic artery chemoembolization, immunotherapy, and targeted therapy on tumor angiogenesis, causing reduced hepatic artery diameter and impaired arterial blood flow. These factors disrupt the liver’s dual blood supply system, ultimately contributing to hepatic infarction.

## Basic information of patients and treatment process

**Fig. 1:**
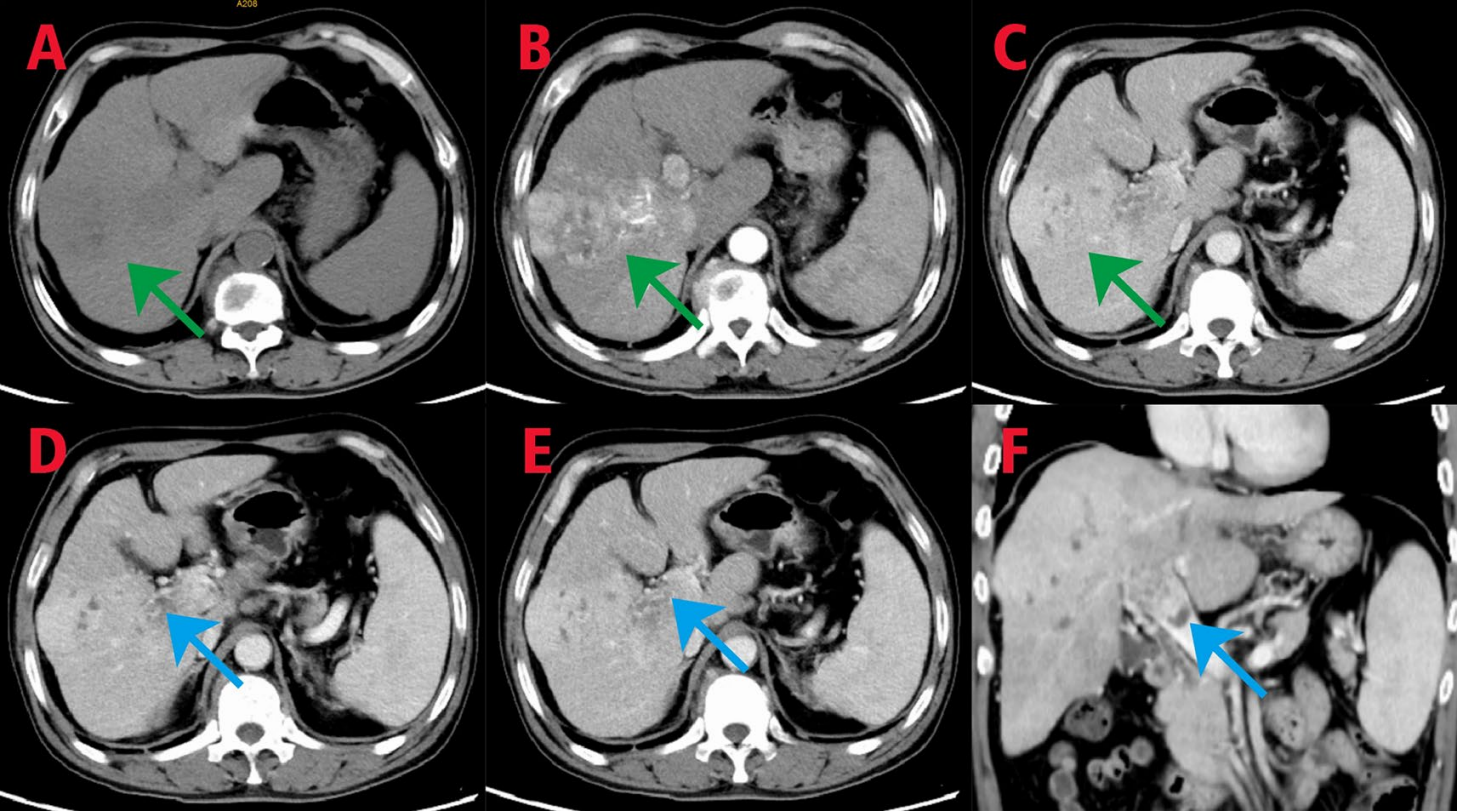
CT imaging findings of the patient upon admission. In the plain scan, a nodular low-density lesion with indistinct borders is observed in the right lobe of the liver **A** indicated by green arrows. The contrast-enhanced scan in the arterial phase shows patchy areas of obvious density and abnormal enhancement **B** indicated by green arrows, while the enhancement degree is reduced in the portal vein phase and delayed phase **C** indicated by green arrows. There is a patchy filling defect in the main trunk of the portal vein, which is enhanced on the contrast-enhanced scan **D, E**, **F**, indicated by blue arrows

**Fig. 2:**
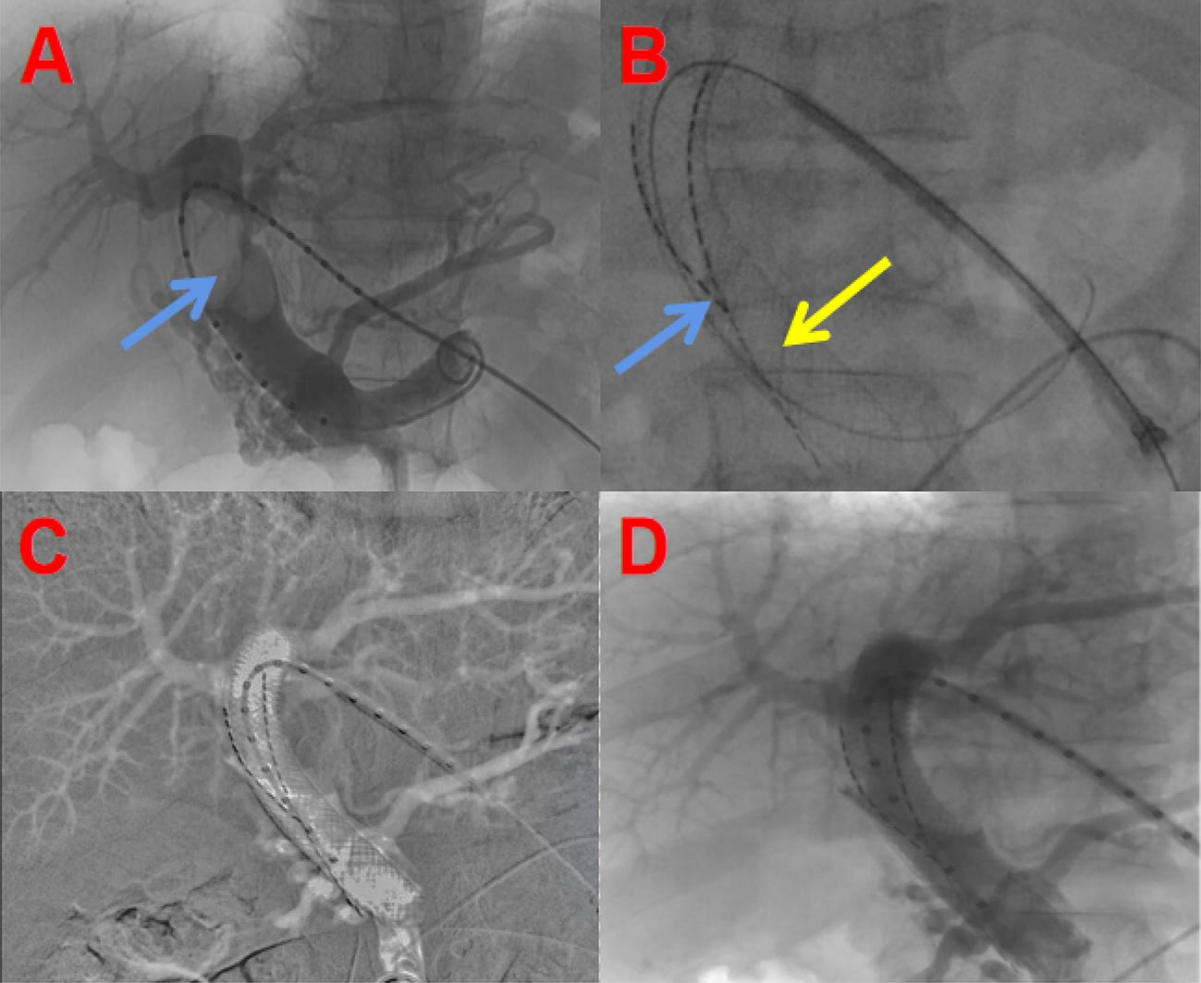
Intraoperative situation of portal vein stent implantation + particle strand implantation: **A** Portal vein tumor thrombus obstruction (blue arrow); **B** Targeted implantation of particle strand (blue arrow) and deployment of the stent (orange arrow); **C** Collapse of the tumor thrombus, with patent portal vein flow; **D** Follow-up angiography showing well-placed stent

In 2022, a 52-year-old male patient was admitted for "black stool for 1 week and discovery of liver occupying lesion for 3 days”. The patient had a history of hepatitis B. Gastroscopy upon admission revealed ruptured esophageal varices, and endoscopic variceal ligation was performed. CT imaging showed an occupying lesion in the right lobe of the liver with portal vein tumor thrombus formation (Fig. 1). The patient was staged as IIIa according to the CNLC staging system, with no indication for surgical resection. Following a multidisciplinary consultation, the patient underwent "portal vein stent placement + ^125^I radioactive particle interventional therapy" in digital subtraction angiography (DSA, GE Healthcare INNOVA3100) on September 16, 2022. During the procedure, a 14–90 mm Wallstent stent and 30 ^125^I particles were successfully implanted (Fig. 2). Postoperative evaluation showed significant improvement in liver function and various indicators compared to preoperative levels.

**Fig. 3:**
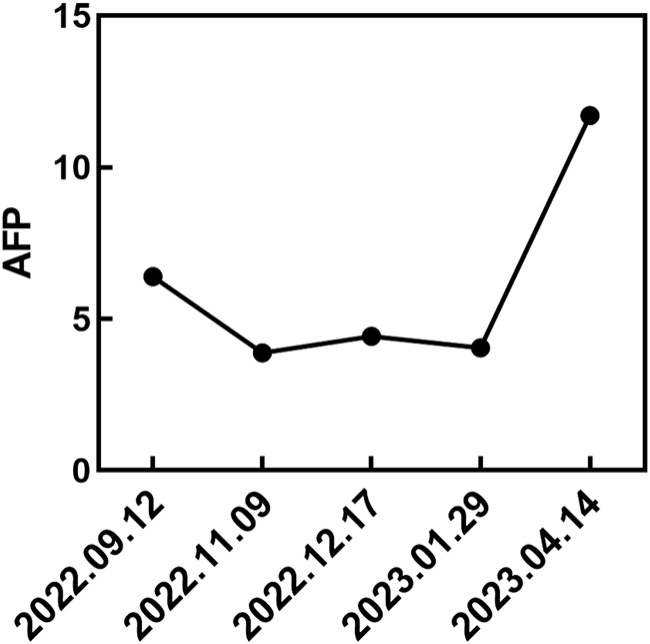
The change of AFP before and after TACE treatment: The AFP level of the patient remained generally within the normal range during the treatment, with a notable increase observed after April

**Fig. 4:**
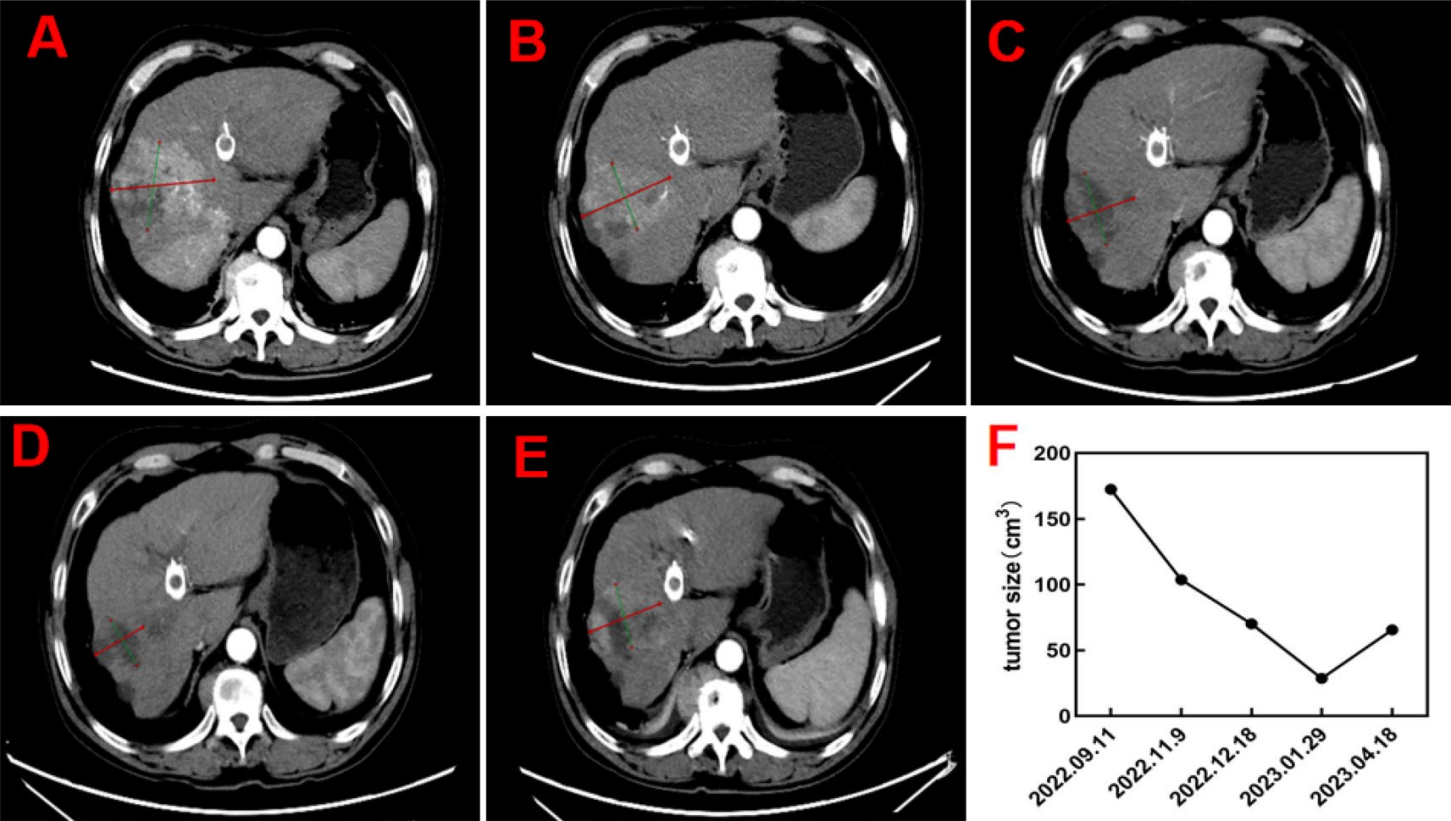
The change size of tumor before and after TACE treatment: CT scans showed pronounced enhancement in the tumor area pre-TACE (**A**), which weakened or disappeared post-TACE treatment (**B, C, D**), and reappeared after 4 months (**E**). The tumor size changes pre- and post-treatment were shown in panel** F**

**Fig. 5:**
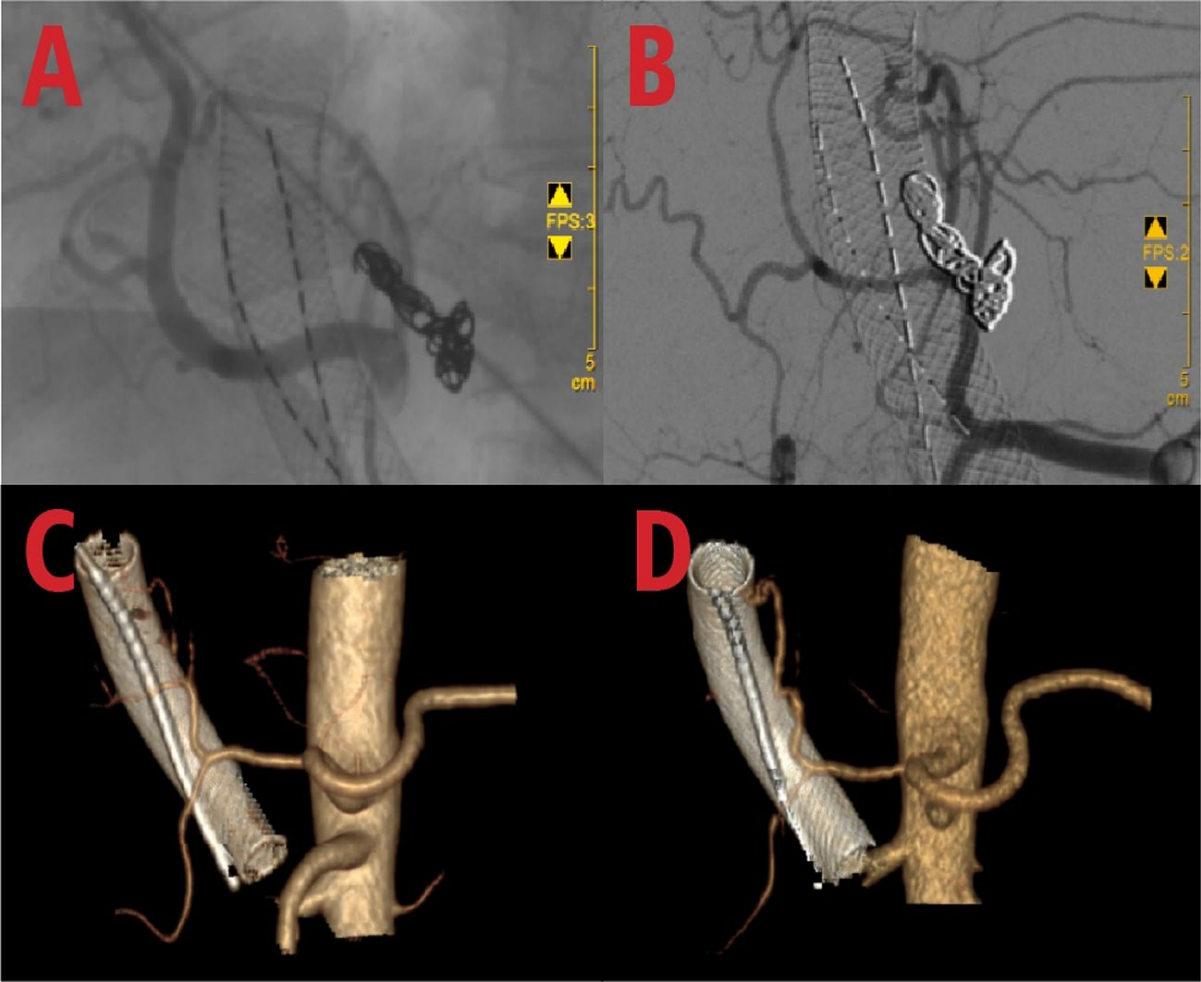
Hepatic artery angiography at 1 months (**A**), 3 months (**B**) and CT scan at 5 months (**C**), 7 months (**D**) after stent implantation showing a gradual decrease in the diameter of the hepatic artery. After stent implantation showing a gradual decrease in the diameter of the hepatic artery

**Fig. 6:**
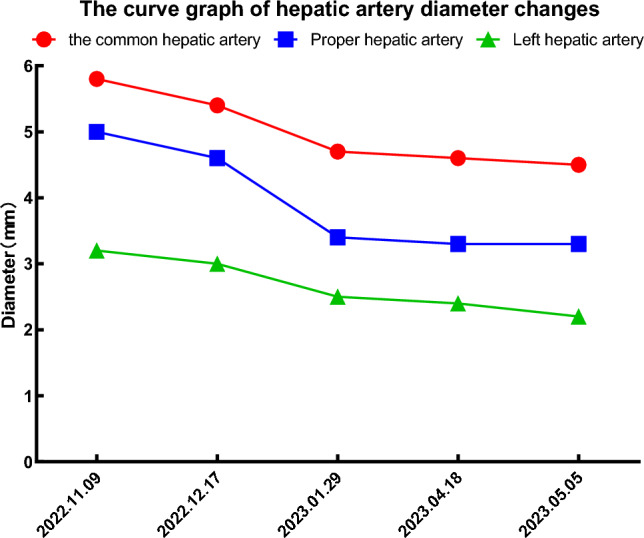
The curve graph of hepatic artery diameter changes demonstrates a gradual reduction in the diameter of the hepatic artery

**Fig. 7:**
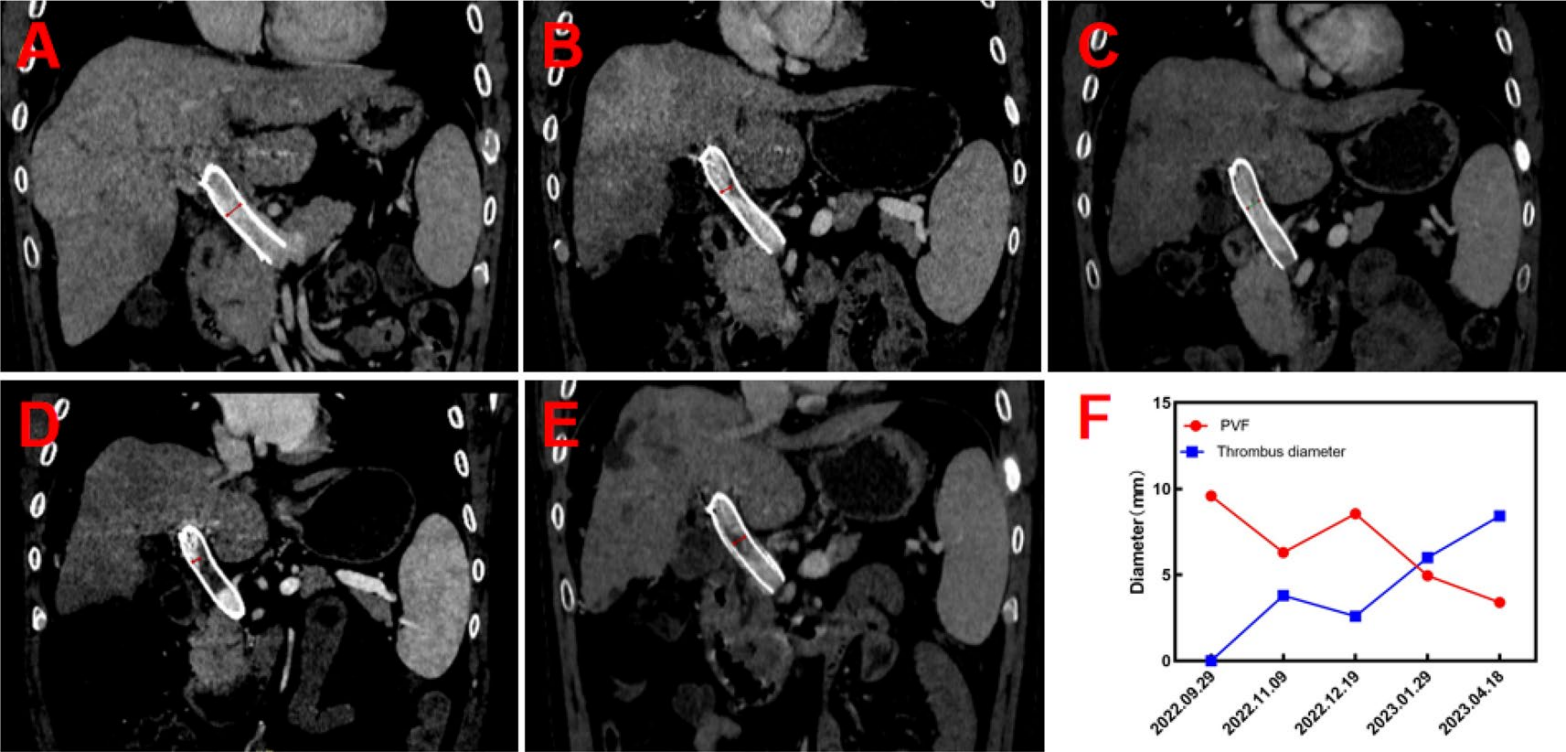
Thrombus formation after stent implantation: No thrombosis was found in the stent 2 weeks after particle stent implantation (**A**). One month after the stent implantation, thrombosis developed in the main portal vein, and blood flow in the portal vein was reduced (**B**). After anticoagulation, portal blood flow increased (**C**) and thrombus was reduced. Repeat examination revealed a gradual increase in stent thrombosis and a decrease in portal vein flow (**D and E**). Changes in portal vein flow and thrombus diameter are shown in panel** F**

**Fig. 8:**
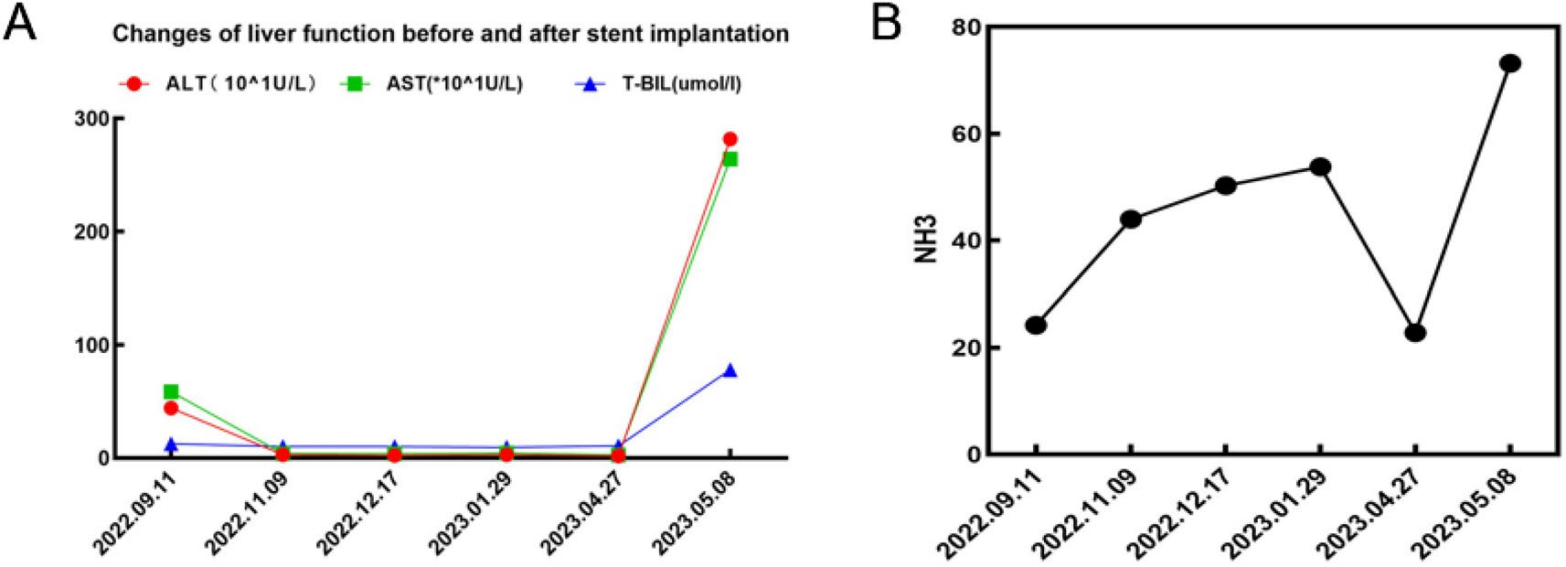
Time-course graph showing changes in liver function and before blood NH3 after stent implantation: Liver function exhibited no significant change pre- and post-TACE treatment, but acute liver failure and hepatic coma (AST, ALT, T-BIL (**A**), NH3 (**B**) increased significantly) occurred, following a 2-month interruption of immune targeted therapy due to gastrointestinal bleeding. After communicating with the patient’s family, they opted to discontinue follow-up treatment, resulting in the patient’s demise one-week post-discharge

**Fig. 9:**
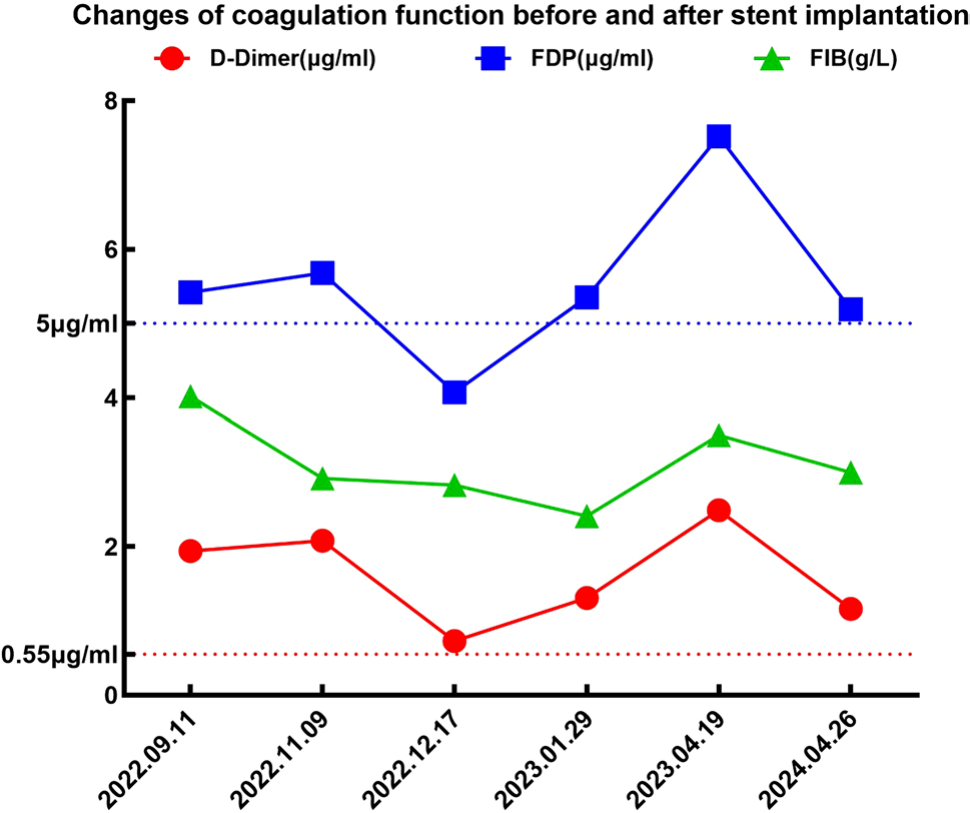
Time-course graph showing changes in coagulation function before and after stent implantation: D-dimer increased and then decreased (red part)

**Fig. 10:**
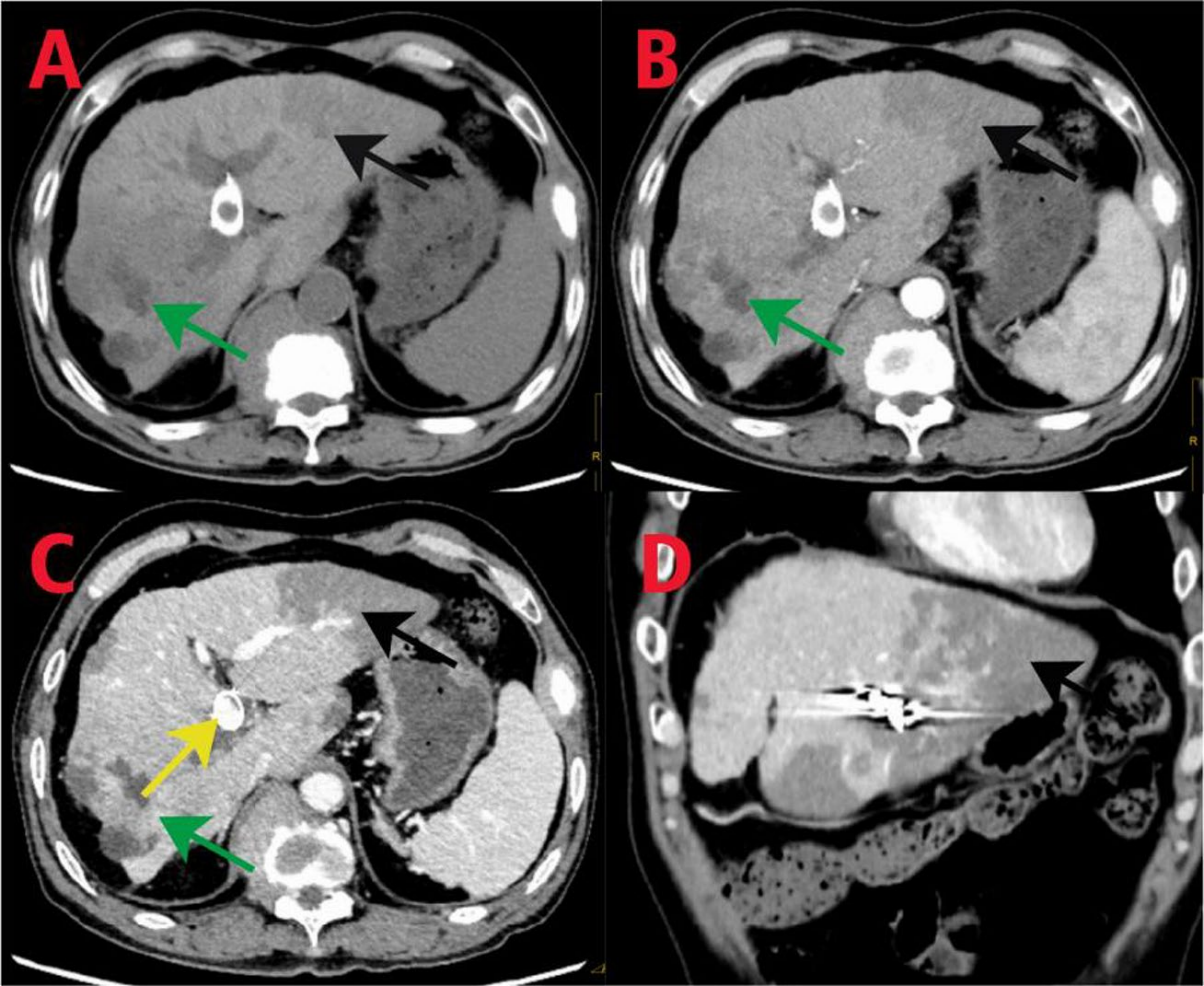
CT imaging findings: In the plain scan, a nodular low-density lesion with indistinct borders is observed in the right lobe of the liver (**A**, green arrow). Contrast-enhanced scan shows no significant enhancement in the arterial phase (**B,** green arrow), and reduced enhancement in the venous phase, with lower enhancement degree compared to the surrounding liver parenchyma. Patchy low-density lesions are visible within the lesion (**C**, green arrow). Multiple patchy and slightly low-density lesions are observed within the liver parenchyma, with no significant enhancement on the contrast-enhanced scan. The previously enhanced lesion within the liver shows no obvious arterial blood supply. A patchy dense shadow is seen in the left lobe of the liver (**A, B, C, D**, black arrow). In the portal vein phase, a crescent-shaped filling defect is observed (reduced compared to previous scans) (**C**, yellow arrow)

In subsequent treatment, the patient received four sessions of hepatic artery chemoembolization (D-TACE): the first time D-TACE was treated with pirarubicin 60 mg-loaded 300–500 μm CalliSpheres®; the second time, gemcitabine 800 mg perfusion + pirarubicin 60 mg-loaded 300–500 μm CalliSpheres®; the third time, pirarubicin 60 mg-loaded 300–500 μm CalliSpheres®; and the fourth time, HepaSphere microspheres 30–60 μm containing pirarubicin 30 mg. Concurrently, immunotherapy (Sintilimab, 200 mg) and targeted therapy (Lenvatinib 8 mg, QD) were combined with primary liver tumor. After treatment, the AFP level of the patient remained generally within the normal range during the treatment, with a notable increase observed after April (Fig. 3).Meanwhile,the patient showed a reduction in the size of the primary liver tumor lesions (By measuring the long and short diameters of the largest tumor area, the tumor size area was calculated for comparison: V_T_ = 1/2(L * W * W) (Du et al. 2017)(Fig. 4), and the diameter of the hepatic artery gradually decreased (Figs. 5, 6). However, during a follow-up CT scan two months after the procedure, thrombus formation was detected within the main trunk of the portal vein stent. Oral administration of 10 mg QD rivaroxaban was promptly initiated for anticoagulant therapy. Subsequent CT scans in March and April revealed an increase in thrombi within the stent compared to before, and an increasing trend in coagulation function (D-dimer and FDP). Six months after the procedure, the patient was readmitted due to "esophageal variceal rupture and bleeding". After anticoagulation, portal blood flow increased and thrombus was reduced. Repeat examination revealed a gradual increase in stent thrombosis and a decrease in portal vein flow (Fig. 7). Endoscopic variceal ligation and pharmacological hemostatic treatment were performed, and bleeding was successfully stopped. Half a month later, the patient was readmitted due to "sudden severe upper abdominal pain". Examination upon admission showed refractory hypotension accompanied by a sharp and progressive elevation in AST, ALT and T-BIL levels (Fig. 8A), blood NH3 increased (Fig. 8B), coagulation function (D-dimer, FDP) decreased (Fig. 9). Abdominal CT scan revealed a decrease in thrombi within the stent compared to previous scans, liver parenchymal necrosis, and the previously enhanced lesion within the liver shows no obvious blood supply (Fig. 10). The patient developed acute liver failure and liver coma. After communicating with the patient’s family, they opted to discontinue follow-up treatment, resulting in the patient’s demise one-week post-discharge.

## Discussion

When primary liver cancer invades the portal vein and forms portal vein tumor thrombus, the thrombus within the main trunk of the portal vein can obstruct portal vein blood flow, leading to portal hypertension. This can result in complications such as esophageal variceal rupture and bleeding, ascites, liver function failure, or even death (Li et al. 2020b; Yuan et al. 2019). Therefore, the prognosis for liver cancer patients with concurrent portal vein tumor thrombus is extremely poor, with a median survival of only 2.7–4 months if left untreated (Villa et al. 2000). Previous reports have shown that portal vein stent implantation combined with ^125^I particle strand implantation is an effective and safe treatment for liver cancer patients with portal vein tumor thrombus. On one hand, they can quickly restore blocked portal vein blood flow, reduce the incidence of portal hypertension, improve liver function, and reduce the risk of esophageal variceal rupture and bleeding. On the other hand, they can directly expose the portal vein tumor thrombus to radiation, thereby controlling tumor progression ((Li et al. 2020a; Yu et al. 2017; Chuan-Xing et al. 2011)). Previous studies have demonstrated that intra-arterial radiation therapy can suppress neointimal hyperplasia after stent implantation and prolong stent patency (Chuan-Xing et al. 2011; Luo et al. 2016). In this reported case, the patient underwent portal vein stent implantation combined with ^125^I particle strand implantation, and the survival period reached 7 months. However, a follow-up CT scan two months after the portal vein stent implantation showed thrombus formation within the stent, and a CT scan at 7 months postoperatively revealed liver parenchymal necrosis, suggestive of hepatic infarction.

Due to the dual blood supply system of the portal vein and hepatic artery in the liver circulation, reports of hepatic infarction are extremely rare, and reports of hepatic infarction following portal vein stent implantation are even rarer. The portal vein supplies approximately 70% of the liver’s blood flow, and when arterial blood supply is insufficient, liver cells can receive oxygen supply through the portal vein (Saegusa et al. 1993; Mayan et al. 2001). There have been reports suggesting a close relationship between the occurrence of hepatic infarction and portal vein thrombosis, indicating that hepatic infarction primarily depends on the disruption of the portal vein system. In addition, circulatory dysfunction can also contribute to inadequate hepatic artery blood supply, further promoting the occurrence of hepatic infarction (Mayan et al. 2001; Lopera et al. 2015). This reported case has its unique aspects. The patient underwent “portal vein stent implantation + ^125^I particle strand implantation,” and a follow-up CT scan two months later revealed thrombus formation within the stent. The patient received anticoagulant therapy after stent thrombosis, and the coagulation function showed a downward trend, but the subsequent CT scan showed an increase in stent thrombosis, accompanied by portal hypertension, and the coagulation function (D-dimer, FDP) increased again. Because the stent is a kind of bare stent, tumor thrombus combined with thrombosis was considered, but thrombosis was the main type. In addition, the patient experienced complications of esophageal varices rupture and bleeding and was treated with variceal ligation and hemostatic medications. The CT scan at 7 months after operation showed that the stent thrombosis decreased, D-dimer and FDP decreased sharply, and the stent thrombosis was considered to fall off. Therefore, our analysis suggests that the main causes of hepatic infarction include: 1. Portal vein stent thrombosis. After anticoagulant therapy, gastroscopic vein ligation, and hemostatic drug treatment, portal hypertension was further aggravated, which affected stent thrombus detachment and caused distal portal vein thromboembolism, thereby blocking portal vein blood flow. After stent placement, the patient underwent multiple transcatheter arterial chemoembolization, immunotherapy, and targeted therapy. These therapies inhibit tumor angiogenesis, leading to a gradual reduction in the diameter of the hepatic artery and affecting hepatic arterial blood flow. The dual blood supply system of portal vein and hepatic artery in hepatic circulation is out of balance, which may contribute to the occurrence of hepatic infarction. Therefore, thrombus formation within the portal vein stent, along with the obstruction of portal vein and hepatic arterial blood flow, plays a crucial role in the development of hepatic infarction following portal vein stent implantation.

Hepatic infarction is a rare complication following portal vein particle stent implantation, and there are currently no reported cases. The case reported in this study represents a typical example of hepatic infarction after portal vein stent implantation, where increased portal vein pressure caused by endoscopic variceal ligation led to thrombus detachment in the portal vein, immunotherapy and targeted therapy led to arterial vascular narrowing resulting in hepatic ischemia. It causes the imbalance of the dual blood supply system of portal vein and hepatic artery. This highlights the importance of guiding the treatment of liver cancer patients with portal vein tumor thrombus undergoing portal vein particle stent implantation.

## Data Availability

No datasets were generated or analysed during the current study.
